# The Functionality of IbpA from *Acholeplasma laidlawii* Is Governed by Dynamic Rearrangement of Its Globular–Fibrillar Quaternary Structure

**DOI:** 10.3390/ijms242015445

**Published:** 2023-10-22

**Authors:** Liliya S. Chernova, Innokentii E. Vishnyakov, Janek Börner, Mikhail I. Bogachev, Kai M. Thormann, Airat R. Kayumov

**Affiliations:** 1 Institute of Fundamental Medicine and Biology, Kazan Federal University, Kremlevskaya 18, 420008 Kazan, Russia; lsch-888@live.com; 2 Institute of Cytology, Russian Academy of Sciences, Tikhoretsky Ave. 4, 194064 St. Petersburg, Russia; innvish@incras.ru; 3 Institute of Microbiology and Molecular Biology, Justus Liebig University, Heinrich-Buff-Ring 26, 35392 Giessen, Germany; janek.boerner@mikro.bio.uni-giessen.de (J.B.); kai.thormann@mikro.bio.uni-giessen.de (K.M.T.); 4 Centre for Digital Telecommunication Technologies, St. Petersburg Electrotechnical University, Professora Popova 5, 197376 St. Petersburg, Russia; rogex@yandex.ru

**Keywords:** *Acholeplasma laidlawii*, heat shock, chaperone, sHSP, IbpA, HSP70, HSP100

## Abstract

Small heat shock proteins (sHSPs) represent a first line of stress defense in many bacteria. The primary function of these molecular chaperones involves preventing irreversible protein denaturation and aggregation. In *Escherichia coli*, fibrillar *Ec*IbpA binds unfolded proteins and keeps them in a folding-competent state. Further, its structural homologue *Ec*IbpB induces the transition of *Ec*IbpA to globules, thereby facilitating the substrate transfer to the HSP70-HSP100 system for refolding. The phytopathogenic *Acholeplasma laidlawii* possesses only a single sHSP, *Al*IbpA. Here, we demonstrate non-trivial features of the function and regulation of the chaperone-like activity of *Al*IbpA according to its interaction with other components of the mycoplasma multi-chaperone network. Our results show that the efficiency of the *A. laidlawii* multi-chaperone system is driven with the ability of *Al*IbpA to form both globular and fibrillar structures, thus combining functions of both IbpA and IbpB when transferring the substrate proteins to the HSP70-HSP100 system. In contrast to *Ec*IbpA and *Ec*IbpB, *Al*IbpA appears as an sHSP, in which the competition between the N- and C-terminal domains regulates the shift of the protein quaternary structure between a fibrillar and globular form, thus representing a molecular mechanism of its functional regulation. While the C-terminus of *Al*IbpA is responsible for fibrils formation and substrate capture, the N-terminus seems to have a similar function to *Ec*IbpB through facilitating further substrate protein disaggregation using HSP70. Moreover, our results indicate that prior to the final disaggregation process, *Al*IbpA can directly transfer the substrate to HSP100, thereby representing an alternative mechanism in the HSP interaction network.

## 1. Introduction

Heat shock proteins (HSPs) play a ubiquitous role in the cell stress response. They protect intracellular proteins from improper folding or aggregation, inhibit signal cascades of apoptosis and preserve intracellular signaling pathways that are necessary for the cell survival [1]. Heat shock proteins are classified by the approximate molecular weights of their subunits as HSP100, HSP90, HSP70, HSP60, HSP40 and the small HSP (sHSP, HSP20) families [2]. Among them, the functional chaperone triad consisting of HSP20, HSP70 and HSP100 is one of the key systems responsible for the reverse protein aggregation in various organisms [3,4,5].

Small heat shock proteins are ATP-independent and represent the first line of protein defense from aggregation. They bind damaged proteins and prevent their further irreversible aggregation; although, they are not capable of further processing the bound proteins [6]. They keep the damaged proteins in a transition state (folding-competent state), from which they can be renaturated into correctly folded forms by ATP-dependent chaperones [7]. Because of their “limited” functionality, they were named also as holdases, unlike foldases, which are responsible for the substrate refolding after stress [8].

Small HSPs are characterized by low molecular weights (12–43 kDa) and exhibit three-domain architecture [9,10,11,12]. Their primary structure consists of a conserved α-crystallin domain (ACD) flanked by a flexible N-terminal region (NTR) of variable length and a short C-terminal region (CTR) [13,14]. The ACD is a structurally compact β-sandwich consisting of two antiparallel sheets of three and four β-strands, typically 80–100 amino acids in length [15]. The NTR generally includes the (W/F)(D/F)PF-like motif, which participates in the oligomerization of sHSPs and is required for their chaperone-like activity [16]. The CTR contains the strongly conserved V/IXI/V-motif, which interacts with the ACD of another subunit in the multimer during oligomerization [17,18,19]. While interacting, the NTR and CTR provide the plasticity of the protein quaternary structure, leading to huge oligomer formation that represents a distinct feature of the sHSPs and provides the basis of their functional activity.

HSP70 is a large ubiquitous family of ATP-dependent molecular chaperones that serve as the central hub of the protein homeostasis system [20,21,22,23]. The ATP-dependent HSP70 protein DnaK is generally involved in the folding of nascent proteins, transport of proteins across membranes, reactivation of improperly folded proteins and control of regulatory proteins activity [24]. DnaK consists of the N-terminal nucleotide-binding domain (NBD) responsible for the ATPase activity, connected via a flexible linker with the C-terminal substrate-binding domain (SBD), which binds hydrophobic polypeptide segments that are exposed in unfolded proteins [21,25,26] (see Supplementary Figure S1a). The binding of nucleotides to the N-terminal domain of DnaK allosterically regulates the interaction of its C-terminal domain with substrates: ADP stabilizes the substrate binding, and ATP triggers its release. In vivo, DnaK interacts with two co-chaperones: DnaJ (HSP40) and GrpE (a nucleotide exchange factor, NEF). DnaJ delivers substrates to DnaK and stimulates ATP hydrolysis, thereby stabilizing DnaK–substrate interactions. GrpE catalyzes the replacement of ADP by ATP bound to DnaK and promotes dissociation of the substrate, thus turning HSP70 to a substrate-binding ready state [27]. Most species possess several other co-chaperones (HSP40 family members and NEFs), which allow DnaK to act on a variety of substrates in various conditions [24].

For further solubilization and reactivation of aggregated proteins, DnaK interacts with HSP100 protein ClpB. ClpB was initially identified as a heat shock protein necessary for the optimal growth and survival of *Escherichia coli* cells at high temperatures [28]. Unlike many other members of the Clp family, which form complexes with peptidase subunits and participate in the protein degradation, ClpB is a member of a multi-chaperone system together with DnaK, DnaJ and GrpE [29,30,31,32,33,34]. The N-terminal domain of ClpB is presumably involved in the substrate uptake from DnaK after DnaJ establishes initial weak contact with the surface of the misfolded protein and transfers a segment of the polypeptide for the interaction with DnaK [35]. ClpB can disaggregate some protein substrates without cooperation with DnaK, although the cooperation of ClpB and DnaK produces the most efficient disaggregation [7,36,37]. Interestingly, the ClpB–DnaK cooperation in protein refolding is species-specific, i.e., ClpB interacts efficiently only with DnaK of the same microorganism [38,39].

The mechanism of the HSP20–HSP70–HSP100 chaperone triad activity is well studied in *E. coli*. Due to the duplication of the original *ibpA* gene, *E. coli* encodes two sHSPs: IbpA (from now on referred to as *Ec*IbpA) and IbpB (from now on referred to as *Ec*IbpB) [40]. *Ec*IbpA and *Ec*IbpB exhibit 48% identity of amino acid sequences while influencing the substrate aggregation process in different ways [41]. *Ec*IbpA binds denatured proteins, tightly forming stable fibril-like aggregates, which eventually become inefficient substrates for their disaggregation by DnaK/DnaJ and ClpB. To initiate refolding, *Ec*IbpA should be displaced from the outer shell of the sHSP–substrate complex, that in turn is facilitated by *Ec*IbpB (Supplementary Figure S2) [40,41,42,43,44,45]. 

The phytopathogenic mycoplasma *Acholeplasma laidlawii* contains only a single sHSP encoding gene named *ibpA* (from now on referred to as *AlibpA*) [46,47]. In *Al*IbpA, the N-terminus provides the formation of 24-mer globules of *Al*IbpA while also behaving as an autoinhibitor and activity regulator of the C-terminus, which in turn is required for the chaperone-like activity and protein oligomerization in the fibrous form [48]. Here, we show that the N-terminus of *Al*IbpA exerts the function of *Ec*IbpB and facilitates further substrate protein disaggregation using HSP70. Moreover, *Al*IbpA seems to directly transfer the substrate to HSP100 prior to the final disaggregation, in marked contrast to the well-described HSP70 from *E. coli*, thereby representing an alternative mechanism in the bacterial HSP interaction network.

## 2. Results

### 2.1. AlIbpA in Active (Substrate-Binding) Form Is Present as Fibrils In Vitro

While *Al*IbpA has 18% identity with *Ec*IbpA and 20% identity with *Ec*IbpB (Supplementary Figure S3A,B), the N-terminal domain appears closer to *Ec*IbpB than to *Ec*IbpA (25% vs. 18% identity), and the C-terminal domain demonstrates 27% identity with *Ec*IbpA and 0% with *Ec*IbpB (Supplementary Figure S3C). Previously, we have shown that while the full-length *Al*IbpA is present mainly as globules with rare fibrils in vitro, the removal of 12 N-terminal amino acid residuals leads to predominant fibrils formation and higher chaperone-like activity [48]. These facts allowed us to hypothesize that the N-terminal domain of *Al*IbpA exerts the function of IbpB.

**Figure 1:**
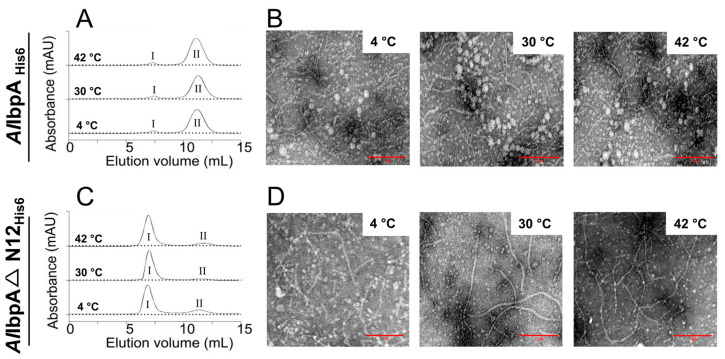
The oligomerization state of recombinant *Al*IbpA at various temperatures. The full-length *Al*IbpA_His6_ and *Al*IbpAΔN12_His6_ were chemically cross-linked at 4 °C, 30 °C or 42 °C and analyzed with size-exclusion chromatography (**A**,**C**) and TEM (**B**,**D**). I (first) and II (second) peaks of the elution profile of *Al*IbpA_His6_.The red scale bar at the bottom right corner of each picture indicates the length of 1 µM.

First, we tested whether these structural changes in *Al*IbpA depend either on the temperature or on the presence of N-termini. For that, full-length *Al*IbpA_His6_ and truncated *Al*IbpAΔN12_His6_ were incubated at different temperatures (4 °C, 30 °C and 42 °C), cross-linked with glutaraldehyde followed by the size-exclusion chromatography performed at 25 °C (Figure 1A,C). 

Regardless of the temperature, the full-length *Al*IbpA_His6_ was eluted in two peaks, the minor one at 7.6 mL and the major one at 11.1 mL (Figure 1A), which were earlier shown to correspond to either fibrillar or globular forms of oligomers, respectively [48]. *Al*IbpAΔN12_His6_ was eluted predominantly as fibrils also independently of the temperature (Figure 1C). Of note, similar elution profiles were observed also for non-cross-linked proteins (Supplementary Figure S4), confirming no cross-linking artifacts. Transmission electron microscopy (TEM) confirmed that the full-length *Al*IbpA_His6_ was the heterogeneous mixture of both globular and fibrous structures (Figure 1B), while *Al*IbpAΔN12_His6_ was present mostly as fibrils (Figure 1D).

**Figure 2:**
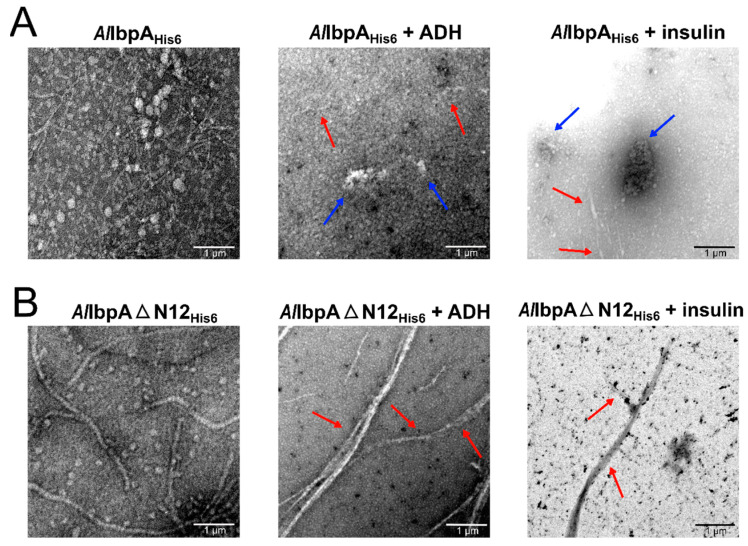
The conformation of oligomers formed by *Al*IbpA_His6_ and *Al*IbpAΔN12_His6_ in presence of substrate proteins. The full-length (**A**) or N-terminally truncated (**B**) *Al*IbpA_His6_ proteins were pre-incubated for 30 min at 25 °C with either ADH (alcohol dehydrogenase) or human insulin, treated with glutaraldehyde and analyzed with TEM. Blue arrows show globular co-aggregates, and red arrows show fibrillar structures. The scale bar at the bottom right corner of each picture indicates the length of 1 µM.

**Figure 3:**
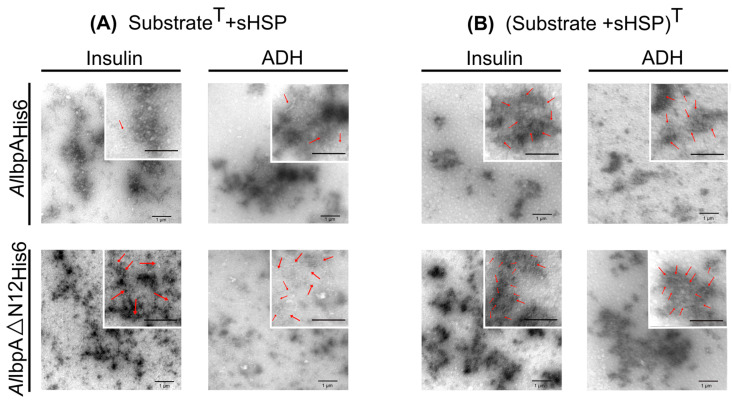
The conformation of oligomers formed by *Al*IbpA_His6_ and *Al*IbpAΔN12_His6_ in presence of substrate proteins under heat-treated alone (**A**) or in the presence of sHSPs (**B**). The substrate proteins were pre-incubated for 30 min at 56 °C (indicated as T) with either *Al*IbpA_His6_ or *Al*IbpAΔN12_His6_, treated with glutaraldehyde and analyzed with TEM. Red arrows show fibrillar structures. The black scale bar at the bottom right corner of each picture indicates the length of 1 µM.

As shown earlier, while being in fibrillar form, *Ec*IbpA tightly interacts with substrate proteins [41], that is in agreement with higher chaperone-like activity of fibrils-forming *Al*IbpAΔN12_His6_ [48]. Therefore, we next studied whether the fibrillar structure of *Al*IbpAΔN12_His6_ is retained after the substrate binding or fibrils represent the “storage” form of the protein. For that, *Al*IbpA_His6_ and *Al*IbpAΔN12_His6_ were mixed with either ADH (alcohol dehydrogenase) or insulin and chemically cross-linked either at 25 °C or at 56 °C, followed by further analysis with TEM (Figure 2 and Figure 3, respectively).

Of note, both insulin and ADH were reported previously as relevant substrates for various HSP20 [49,50,51], including *Al*IbpA [47,48]. In the presence of substrate proteins, at low temperatures, the full-length *Al*IbpA_His6_ led to the formation of aggregates that appeared as globules up to ten-fold larger compared to solely sHSPs or substrate proteins (Figure 2A and Supplementary Figure S5, respectively). By contrast, the *Al*IbpAΔN12_His6_ appeared as fibrils and formed fibrillar structures also in the presence of either ADH or insulin (Figure 2B), despite proteins interacting even at 25 °C, as shown earlier in [48].

The pre-denaturation of either ADH or insulin at 56 °C resulted in the formation of large aggregates (Supplementary Figure S5), and the addition of sHSPs did not change the size and morphology of substrate aggregates (Figure 3A). Samples with the full-length *Al*IbpA_His6_ were of similar conformation as in the experiment performed at 25 °C, primarily forming globule-like structures around the denatured protein; nevertheless, some fibrils were also found near the substrate proteins (see red arrows). *Al*IbpAΔN12_His6_ was observed in the form of fibrils, although they were shorter compared to the low-temperature experiment (compare Figure 2A and Figure 3A). The heat treatment of substrate proteins in the presence of sHSPs led to a different type of aggregate formation, with the aggregates appearing much smaller in their sizes and being more sparsely represented. A large amount of material appeared as short fibrils (50–100 nm in length) surrounded by a contrast agent for both *Al*IbpA_His6_ and *Al*IbpAΔN12_His6_ (Figure 3B), apparently representing substrate–sHSP complexes. 

Taken together, these data suggest that in vitro *Al*IbpA_His6_ transforms from globules to fibrils in the active (substrate-binding) state similarly to *Ec*IbpA.

### 2.2. AlIbpA Interacts with AlDnaK and AlClpB In Vitro

To test whether *Al*IbpA interacts with *Al*DnaK and *Al*ClpB, co-elution of recombinant *Al*IbpA_His6_ on Ni-NTA Sepharose was performed. *Al*IbpA_His6_ was pre-incubated with extracts of *A. laidlawii* cells at either optimal (30 °C) or stress conditions (4 °C and 42 °C) and purified. Those proteins that co-eluted with the recombinant *Al*IbpA_His6_ were identified by LC–MS (liquid chromatography–mass spectrometry). In addition to a broad spectrum of other proteins, *Al*ClpB and *Al*DnaK were identified regardless of the incubation temperature (Table 1). However, more HSPs were co-eluted with increasing temperature, as indicated by higher emPAI values at 42 °C. 

The alignment of *dnaK* and *clpB* genes from *A. laidlawii* and *E. coli* revealed 54% and 56% identity, respectively (Supplementary Figures S1 and S6), suggesting similarities in the HSP20–HSP70–HSP100 systems functionality in these bacteria. 

**Figure 4:**
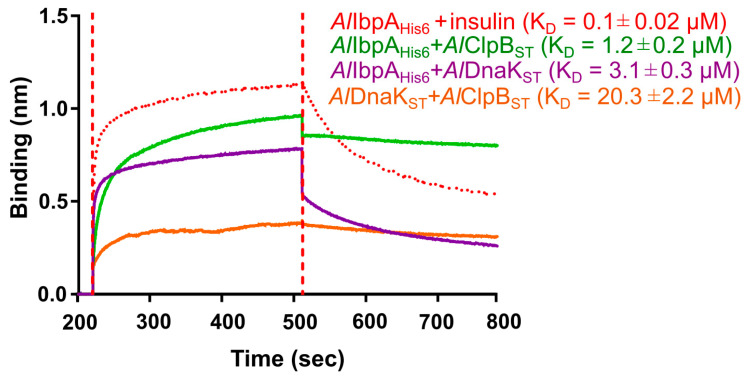
The interferometric analysis of recombinant *Al*IbpA, *Al*ClpB and *Al*DnaK interaction in vitro on the BLItz instrument (FortéBio, Fremont, CA, USA). Either *Al*IbpA_His6_ or *Al*DnaK_ST_ was loaded onto a streptavidin biosensor until 2.0 nm protein was bound to the surface, and after 30 s washing in PBS, the sensor was immersed into either *Al*ClpB_ST_ or *Al*DnaK_ST_ solutions (10 µM). The dissociation constant (K_D_) calculation was performed with BLItz Pro data analysis software (version: 1.3.0.5). The interaction of *Al*IbpA with insulin (red dots) served as a positive binding control. The standard deviations are indicated as range behind the calculated mean values of technical triplicates.

*Al*DnaK_ST_ and *Al*ClpB_ST_ were overexpressed in *E. coli*, purified, and the interaction of the recombinant *Al*IbpA_His6_ with *Al*DnaK_ST_ and *Al*ClpB_ST_ was studied in vitro with bio-layer interferometry using the BLItz system (Figure 4). Both *Al*DnaK_ST_ and *Al*ClpB_ST_ were able to interact with *Al*IbpA_His6_ (dissociation constant K_D_ = 3.1 ± 0.3 μM and 1.2 ± 0.2 μM, respectively), although less efficiently compared to insulin (K_D_ = 0.1 ± 0.02 μM). Interestingly, the *Al*IbpA_His6_–*Al*ClpB_ST_ complex became very stable as dissociation was low. The interaction between *Al*DnaK_ST_ and *Al*ClpB_ST_ was weak (K_D_ = 20.3 ± 2.2 μM), suggesting that these proteins interact preferentially with sHSPs. Furthermore, the interaction of the recombinant *Al*IbpA_His6_ with *Al*DnaK_ST_ or *Al*ClpB_ST_ was confirmed via co-elution on the agarose Ni-NTA column (Supplementary Figure S7).

### 2.3. The Impact of AlIbpA in Fibrillar Form on Its Interaction with AlDnaK and AlClpB and Substrate Transfer to HSPs

It was shown earlier that *Ec*IbpA bound in fibrillar form to the substrate protein impairs the transfer of the latter to DnaK and ClpB [40,41,42,43,44,45]. Therefore, we studied the impact of *Al*IbpA in fibrillar form for the interaction with *Al*DnaK and *Al*ClpB and substrate transfer to HSPs in vitro and in vivo. 

**Figure 5:**
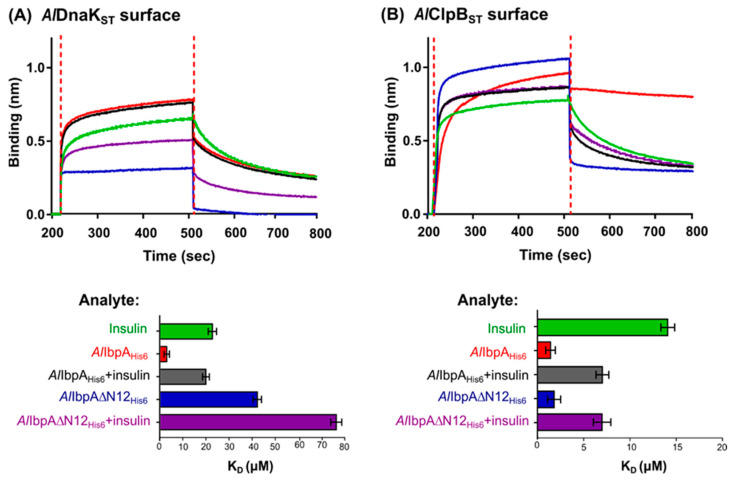
The interferometric analysis of recombinant *Al*DnaK and *Al*ClpB interaction with sHSP-bound substrate in vitro on the BLItz instrument (FortéBio, Fremont, CA, USA). Either *Al*DnaK_ST_ (**A**) or *Al*ClpB_ST_ (**B**) was loaded onto streptavidin biosensor until 2.0 nm protein was bound to the surface. After 30 s washing in PBS, the sensor was immersed into recombinant either full-length *Al*IbpA_His6_ or *Al*IbpAΔN12_His6_ solely protein solution or proteins chemically cross-linked with insulin by glutaraldehyde. K_D_ calculation was performed with BLItz Pro data analysis software (version: 1.3.0.5). The standard deviations of the mean values from technical triplicates are shown as error bars.

Our results indicate that the affinities of full-length *Al*IbpA_His6_ to the surface-bound *Al*DnaK_ST_ and *Al*ClpB_ST_ were similar to previously reported data (K_D_ = 0.1 μM and 0.2 μM, respectively, comparing Figure 4 and Figure 5). The deletion of the N-terminus of *Al*IbpA decreased the binding affinity of *Al*IbpA∆N12_His6_ to *Al*DnaK_ST_ 15-fold (Figure 5A), suggesting that the fibrillar form of *Al*IbpA impairs its interaction with large HSPs, as shown previously for fibrillar *Ec*IbpA. In contrast, no similar effect could be observed for *Al*ClpB_ST_, assuming no significant impact of the *Al*IbpA quaternary structure on the interaction of these proteins.

Next, the interaction of *Al*DnaK and *Al*ClpB with insulin complexes with either fibrillar or globular forms of *Al*IbpA in sHSP–substrate complexes (as seen in Figure 2) was investigated. For that, either *Al*IbpA_His6_–insulin or *Al*IbpA∆N12_His6_–insulin complexes were cross-linked in vitro. Further, their interaction with either *Al*ClpB_ST_ or *Al*DnaK_ST_ immobilized on the sensor was analyzed. 

Affinities of both *Al*DnaK_ST_ and *Al*ClpB_ST_ to full-length *Al*IbpA_His6_ bound with insulin increased approximately five-fold compared with solely sHSPs (Figure 5), suggesting that *Al*IbpA dissociation from the complex with substrate protein is required to recruit the latter to bind with the large HSPs, as shown previously in *E. coli* [45]. Of note, only a two-fold increase in K_D_ was observed for *Al*DnaK_ST_ interaction with insulin-bound *Al*IbpA∆N12_His6_, suggesting that the fibrillar form of sHSPs appears as the main negative factor affecting the protein interaction. These data suggest that the interaction of *Al*IbpA in fibrillar form with *Al*DnaK is unfavorable, although the interaction with *Al*ClpB does not depend on the sHSP quaternary structure.

**Figure 6:**
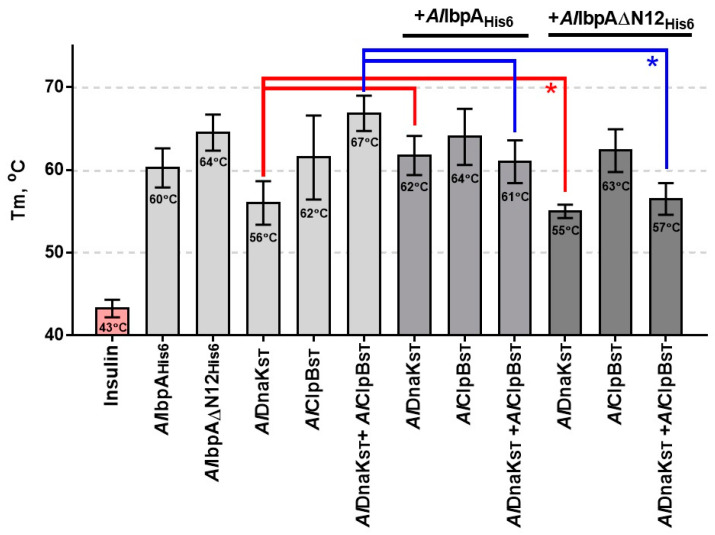
The capacity of recombinant *Al*HSPs to prevent the temperature-induced denaturation of insulin in vitro. Various recombinant *Al*HSPs (0.1 mg) were mixed with insulin (0.1 mg) in total volume of 30 μL and heated from 10 °C to 95 °C with a temperature increment of 1 °C per 1 min in the presence of 10 μM SYPRO Orange. The fluorescence was detected using FAM filter set detection on Bio-Rad CFX96 thermocycler. The data analysis was performed by determining the temperature leading to fluorescence increase by 100% of maximal signal (Tm). Asterisks show statistically significant differences (*p* < 0.05). Error bars indicate the standard deviation of the mean values from technical triplicates.

Further, the role of both fibrillar and globular forms of oligomeric *Al*IbpA for the cooperation of the latter with HSP70 and HSP100 was evaluated via assessing the ability to prevent the insulin denaturation in vitro. Human insulin was heated in the presence of various combinations of HSP20, HSP70 and HSP100, as indicated in Figure 6, and the melting temperature (Tm) was assessed using SYPRO Orange assay. The Tm values of pure proteins are shown in Supplementary Figure S8.

Solely insulin was denatured completely at 43 °C; whereas, in the presence of either full-length or truncated *Al*IbpA_His6_, the Tm increased up to 60 °C and 64 °C, respectively. *Al*DnaK_ST_ and *Al*ClpB_ST_ alone were less efficient in the insulin denaturation prevention (Tm = 56 °C and 62 °C, respectively) compared to their complex (Tm = 67 °C). The combination of *Al*DnaK_ST_ with *Al*IbpA_His6_ increased the Tm by 6 °C (*p* < 0.05), while *Al*IbpA∆N12_His6_ abrogated this effect (see Figure 6 red line). The combination of either *Al*IbpA_His6_ or *Al*IbpA∆N12_His6_ with *Al*ClpB_ST_ did not affect the Tm. The addition of either *Al*IbpA_His6_ or *Al*IbpA∆N12_His6_ to the *Al*DnaK_ST_–*Al*ClpB_ST_ complex led to Tm to decrease by 6 °C and 10 °C, respectively. Neither *Al*IbpA_His6_ nor *Al*IbpA∆N12_His6_ affected the Tm of insulin in the presence of *Al*ClpB_ST_, which is in agreement with their close binding affinities (see Figure 5B).

### 2.4. The In Vivo Assessment of the Role of HSP20, HSP70 and HSP100 in Heat Resistance of Cells

**Figure 7:**
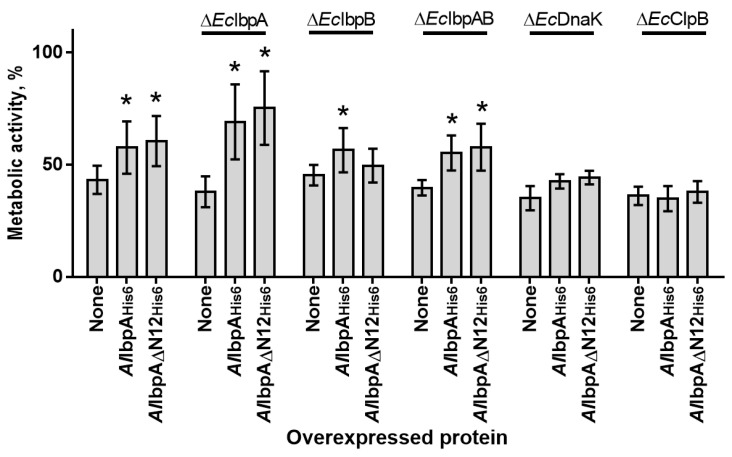
The viability of *E. coli* BL21 and its HSP-deficient derivatives ectopically overexpressing either full-length or N-terminally truncated *Al*IbpA. Cells carrying pET15b vector without insert served as a control (marked as “None”). Exponentially growing cells were induced with 1 mM IPTG. After 2 h of growth at 30 °C, cultures were heat-treated (1 h at 56 °C) and residual viability was assessed in MTT assay. Data are present as mean ± standard deviations of biological triplicates with 3 technical repeats of each. The metabolic activity of cells growing at 37 °C was considered as 100%. Asterisks show statistically significant difference between cell with and without sHSP overexpression (*p* < 0.05). The standard deviations of the mean from biological triplicates are shown as error bars.

The effect of *Al*IbpA_His6_ and *Al*IbpA∆N12_His6_ overexpression on the heat resistance was evaluated in recombinant *E. coli* BL21 cells lacking their own endogenous HSPs. *E. coli* strains carrying pET-*Al*IbpA_His6_, pET-*Al*IbpA∆N12_His6_ and pET15b empty vector as negative control were induced with the addition of IPTG and after 1 h incubation at 30 °C were subjected to heat treatment (56 °C) for 1 h. The cellular viability was measured using the MTT test (Figure 7) and expressed as a percentage of the residual metabolic activity of the cells after heating. Additionally, colony forming units (CFUs) were calculated. These data are shown in Supplementary Figure S9.

The thermotolerance of *E. coli* did not decrease significantly upon deletion of physiological HSPs indicated by comparable residual viability of all strains (40–50%). The heterologous expression of full-length *Al*IbpA_His6_ increased the thermotolerance of Δ*Ec*IbpA and in lesser extent of Δ*Ec*IbpB and Δ*Ec*IbpAB cells, while *Al*IbpAN12_His6_ affected only Δ*Ec*IbpA and Δ*Ec*IbpAB cells. These data suggest that the N-terminus of *Al*IbpA is responsible for the same functions as *Ec*IbpB. No effect of either *Al*IbpA_His6_ or *Al*IbpAN12_His6_ on the thermotolerance of Δ*Ec*DnaK and Δ*Ec*ClpB cells could be observed, suggesting that *Al*IbpA could replace own sHSPs of *E. coli* in Δ*Ec*IbpA, Δ*Ec*IbpB and Δ*Ec*IbpAB cells.

## 3. Discussion

HSPs are cellular tools participating in protein quality control by the refolding of misfolded proteins [1]. A functional chaperone triad consisting of HSP20, HSP70 and HSP100 is one of the main systems responsible for the damaged protein refolding [3,4,5].

Small heat shock proteins bind damaged proteins, prevent their further irreversible aggregation and transfer them to ATP-dependent chaperones that are capable of renaturating them into correctly folded forms [7]. A crucial function of the sHSPs–substrate interaction is keeping the aggregating proteins in a refolding-competent state, which prevents their irreversible denaturation and subsequently allows refolding by HSP70 and HSP100 [7]. The sHSP system of *E. coli*, consisting of the two proteins *Ec*IbpA and *Ec*IbpB, is still considered the most studied among prokaryotes (Supplementary Figure S2) [40]. In the form of fibril-like oligomers, *Ec*IbpA efficiently binds proteins with disrupted conformation. In complex with *Ec*IbpB, the fibrillar *Ec*IbpA forms globule-like oligomers that can easily transfer the substrate to the next members of the chaperone system by dissociating from the substrate (Supplementary Figure S2a). However, the absence of *Ec*IbpB disrupts this process, complicating further interaction of *Ec*DnaK with the substrate and thereby inhibiting substrate processing by the multi-chaperone system (Supplementary Figure S2b) [43,44,52]. 

*Al*IbpA forms in vitro a mixture of fibrillar and globular oligomers suggesting that it possesses the function of both IbpA and IbpB (Figure 1A,B [48]). The deletion of the N-terminus of *Al*IbpA led to the formation of homogenous fibrillar quaternary protein structure depending neither on the temperature (Figure 1C,D) nor on the substrate binding (Figure 2B). This fact allows for the suggestion that the N-terminus of *Al*IbpA could possess the function of *Ec*IbpB from the *E. coli* dual-sHSP system (Supplementary Figure S2). Further, the substrate proteins treated with high temperature (56 °C) in the presence of sHSPs formed complexes, appearing as short fibrils for both *Al*IbpA_His6_ and *Al*IbpAΔN12_His6_ (Figure 3B). Thus, both fibrillar and globular *Al*IbpA_His6_ are capable of dynamically rearranging into short fibrils to exhibit chaperone-like activity towards the aggregating substrate (Figure 3B). Together with higher chaperone-like function of fibrillar *Al*IbpAΔN12_His6_ compared to globular *Al*IbpA_His6_ (Figure 6, [48]), it is likely that the fibrillar quaternary structure represents the active form of *Al*IbpA. Thus, based on the quaternary structure similarities of the N-terminal-truncated *Al*IbpA_His6_ (Figure 2B) and *Ec*IbpA [53], we assume that *Al*IbpA behaves as *Ec*IbpA, which efficiently binds denatured proteins by forming fibril-like co-aggregates. By contrast, *Ec*IbpA is inefficient for DnaK/DnaJ/GrpE- and ClpB-mediated disaggregation in the absence of *Ec*IbpB because of its inability to transit to globular form and dissociate from the substrate [40,42,43,44,45]. Indeed, when insulin was chemically bound to either *Al*IbpA_His6_ or *Al*IbpAΔN12_His6_, the interaction of these complexes with both *Al*DnaK_ST_ and *Al*ClpB_ST_ decreased drastically (Figure 5), probably due to the competitive substitution of sHSPs by large HSPs becoming unavailable. Furthermore, *Al*IbpAΔN12_His6_ had 15-fold lower affinity to *Al*DnaK_ST_ (Figure 5A), apparently because of its tight binding to insulin in fibrillar form, behaving similar to *Ec*IbpA. Unlike *Ec*ClpB, the interaction of *Al*IbpAΔN12_His6_ with *Al*ClpB_ST_ was not affected (Figure 5B). 

On the other hand, *Al*IbpAΔN12_His6_ was not only impaired in binding to *Al*DnaK_ST_ (Figure 5A) but also the activity of the entire multi-chaperone system was repressed, as shown by the in vitro chaperone activity assay (Figure 6). A similar effect was described for multi-chaperone activity of *E. coli* in *Ec*IbpB-deficient cells (Supplementary Figure S2B, [41]). Thus, *Al*IbpA_His6_ had no influence on in vitro chaperone activity in the presence of one component of the HSP70–HSP100 system (Figure 6). When both HSPs were present, the functionality of this system decreased by the presence of *Al*IbpA_His6_ due to incomplete sHSP–substrate complex dissociation. Despite the ability of *Al*ClpB_ST_ to interact with the *Al*IbpAΔN12_His6_–substrate complexes (Figure 5B), the presence of *Al*DnaK_ST_ seems to inhibit the multi-chaperone activity in vitro, lining up that *Al*IbpA interacts with *Al*DnaK prior to *Al*ClpB (Figure 6).

To evaluate the physiological role of HSP20, HSP70 and HSP100 from *A. laidlawii* in vivo, an artificial recombinant system with *E. coli* BL21 was used, where the *ibpA*, *bpB*, *dnaK* and *clpB* genes were deleted. *E. coli* cells expressing full-length *Al*IbpA_His6_ became thermotolerant to high temperature (56 °C), while retaining the growth comparable to that observed at 37 °C (Supplementary Figure S10D), suggesting that *Al*IbpA is able to fulfill beneficial functions in the early heat stress response of this organism, as it was already shown when overexpressing native IbpA or IbpB [54]. Remarkably, a similar phenotype was observed for the *dnaK* deletion mutant, expressing *Al*IbpA_His6_, even though the biological triplicates showed higher heterogeneity in the growth behavior. Without *Al*IbpA_His6_ overexpression, no growth could be observed at 56 °C, which further emphasizes the inhibitory effect on growth of *E. coli* BL21 at this temperature (Supplementary Figure S10B). 

The thermotolerance of *E. coli* cells did not strongly decrease when the endogenous sHSPs were deleted (Figure 7, Supplementary Figure S10). The overexpression of full-length *Al*IbpA_His6_ and the N-terminal-truncated *Al*IbpA∆N12_His6_ were able to compensate the deficiency of either *Ec*IbpA or both *Ec*IbpAB, but not *Ec*IbpB (Figure 7), suggesting that *A. laidlawii* N-terminus is responsible for the IbpB-like protein functions. Of note, truncated *Al*IbpA increased the thermotolerance stronger than the full-length *Al*IbpA_His6_, which could be due to its constitutive fibrillar structure. 

**Figure 8:**
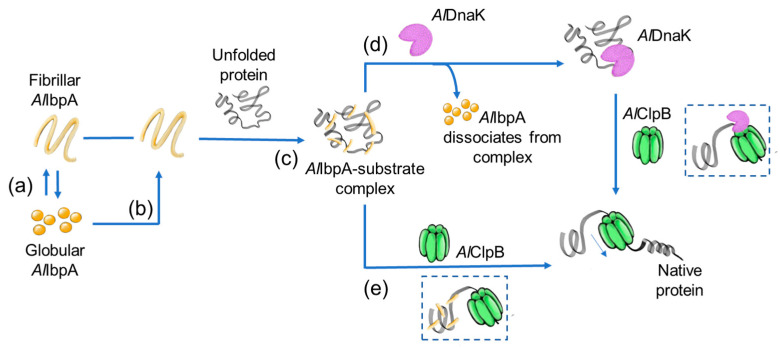
The proposed model of HSP20–HSP70–HSP100-mediated protein disaggregation mechanism in *A. laidlawii*. Inactive *Al*IbpA is a heterogeneous mixture of globular and fibrillar oligomers (a). During disaggregation, globular *Al*IbpA reorganizes into fibril-like oligomers (b), which efficiently bind proteins with disrupted conformation (c). Stably bound to the substrate, *Al*IbpA, in the form of short fibrils (active form of *Al*IbpA), is able to dissociate from the *Al*IbpA–substrate complex in the presence of *Al*DnaK for further refolding of the substrate by the *Al*DnaK–*Al*ClpB chaperone system (d) or directly transfer the substrate to *Al*ClpB (e).

Taken together, our data demonstrate non-trivial features of the function and regulation of the chaperone-like activity of sHSP *Al*IbpA from *A. laidlawii*. Previously, we reported [48] that the N-terminal domain of *Al*IbpA provides the formation of 24-mer *Al*IbpA globules and acts as an autoinhibitor and regulator of the C-terminal domain, which is necessary for the chaperone-like activity and oligomerization of the protein into a fibrillar form. Thus, the effectiveness of the *A. laidlawii* multi-chaperone system seems to be based on the ability of *Al*IbpA to form both globular quaternary structures, which are necessary for the sHSP–substrate dissociation as well as on the subsequent protein disaggregation, and fibrils, which, in contrast to *Ec*IbpA, do not inhibit the system in vivo (Figure 8). In contrast to *Ec*IbpA and *Ec*IbpB of *Escherichia coli*, which regulate its activity in close cooperation, to the best of our knowledge, *Al*IbpA appears the first sHSP, in which the competition between the N- and C-terminal domains regulates the shift of the quaternary structure of the protein into either fibrillar or globular form, thus representing a molecular mechanism of the regulation of its function. Based on our results, we postulate that prior to the final disaggregation process, *Al*IbpA can directly transfer the substrate to HSP100, in contrast to the well-described HSP70 from *E. coli*, thereby representing an alternative mechanism in the HSP interaction network. 

## 4. Materials and Methods

### 4.1. Protein Alignment and Structure Analyses 

The multiple alignment of amino acid sequences was performed using Clustal Omega web server [55] (accessed on 10 July 2023) and manually refined. The sHSP sequences and models of tertiary structures were obtained using Uniprot servers (accessed on 10 July 2023): *Al*IbpA from *Acholeplasma laidlawii* PG-8A WP_012242373.1, *Ec*IbpA from *Escherichia coli* K12 WP_053890241.1 and *Ec*IbpB from *Escherichia coli* K12 NC_000913.3.

### 4.2. Strains and Plasmids 

*E. coli* XL-10 Gold (Agilent, Santa Clara, CA, USA) was used for cloning procedures. *E. coli* BL21 (Agilent, Santa Clara, CA, USA) and its derivatives were used for protein overexpression and in vivo experiments. Primers and plasmids used in this study are listed in Supplementary Tables S1 and S2, respectively. Synthetic genes *dnaK* and *clpB* encoding the HSP70 and HSP100 from *A. laidlawii*, respectively, were synthesized and cloned in pET-28a(+) vector linearized with the restriction endonucleases *Xba*I and *Bam*HI by Synbio Technologies (Monmouth Junction, NJ, USA). The resulting plasmids pET-28a-*Al*DnaK_ST_ and pET-28a-*Al*ClpB_ST_ provided the IPTG-inducible expression of recombinant C-terminally StrepII-tagged (ST) *Al*DnaK and *Al*ClpB, respectively, in *E. coli* BL21 cells. 

The deletion of HSP genes in *E. coli* BL21 cells was performed accordingly to the protocol described previously by [56]. The *ibpA*, *ibpB* and whole *ibpAB* operon were replaced by a kanamycin resistance cassette; *dnaK* and *clpB* were replaced by spectinomycin and chloramphenicol resistance cassettes, respectively. The deletion schemes are shown in Supplementary Figure S11, and primers are listed in Supplementary Table S1. 

### 4.3. Protein Purification 

All recombinant proteins were overexpressed in *E. coli* BL21 (DE3) (Stratagene, La Jolla, CA, USA). The His6-tagged proteins were purified on Ni-NTA agarose (Qiagen, Hilden, Germany), as described in [48]. StrepII-tagged proteins were purified on Strep-tactin agarose (Qiagen, Hilden, Germany), as described in [57]. Detailed description of procedures is given in Supplementary Materials. All proteins were purified to apparent electrophoretic homogeneity, dialyzed against PBS pH 7.4 containing 100 mM NaCl and used immediately for experiments or stored at 4 °C no more than 4 days.

### 4.4. In Vitro Cross-Linking of Proteins 

The chemical cross-linking using glutaraldehyde (GA) was performed as described in [48]. *Al*IbpA_His6_ proteins (1.0 mg mL^−1^) were mixed with human insulin (Sigma-Aldrich, St. Louis, MO, USA) (1.0 mg mL^−1^), and after 10 min incubation at either 25 °C or 56 °C, the mixture was treated with 0.05% (*v*/*v*) freshly prepared solution of glutaraldehyde. The reaction was stopped by the addition of 100 mM Tris pH 7.4 after 20 min of incubation.

### 4.5. Size-Exclusion Chromatography 

The analytical size-exclusion chromatography was performed on Superdex 200 10/300 column (Sigma-Aldrich, St. Louis, MO, USA) at flow rate of 0.5 mL min^−1^ on Waters Breeze 2 equipment with the absorbance detection at 280 nm. The protein sample (1 mg mL^−1^) was centrifuged for 5 min at 12,000 rpm to remove any sediment, and 100 μL were injected. The running buffer contained 50 mM NaCl, 50 mM KCl and 50 mM K2HPO4 pH 7.4. Apparent molecular weights of proteins were estimated after column calibration with blue dextran (2000 kDa), β-amylase (200 kDa), alcohol-dehydrogenase (147 kDa), BSA (66 kDa), papain (23.4 kDa) and lysozyme (14.3 kDa).

### 4.6. Pull Down Assay 

The interaction of purified proteins was assessed on Ni-NTA Sepharose or Strep-Tactin Sepharose (IBA Lifesciences, Göttingen, Germany) on 0.05 mL columns. The purified proteins were mixed in 1:1 (*w*/*w*), incubated for 1 h at room temperature with shaking in the presence or absence of ligands. Afterwards, 50 µL of resin was added to the samples and incubated for 30 min. Samples were loaded onto columns, washed with 2 mL of His-Wash or Strep-Wash buffer and eluted with 1 mL of His-Elution or Strep-Elution buffer (see Supplementary Materials data for composition). Proteins from the elution fractions were precipitated with trichloroacetic acid and analyzed using SDS-PAGE, followed by silver staining.

To identify potential partner proteins for interaction, the purified recombinant protein was co-eluted with bacterial cell extract on 0.5 mL columns.

*A. laidlawii* cells were grown in liquid-complete Edwardian nutrient medium (ENM) until early stationary growth phase, harvested by centrifugation, and cell extract was prepared at 30 °C. A total of 1 mg of purified *Al*IbpA_His6_ was added to the cell extract, followed by incubation for 1 h at 4 °C, 30 °C, 37 °C or 42 °C. Afterwards, samples were loaded onto columns containing 0.5 mL of Ni-NTA Sepharose, washed with His-Wash buffer and eluted with His-Elution buffer containing 250 mM imidazole. The eluate was harvested, precipitated with trichloroacetic acid and analyzed using SDS-PAGE, and co-eluted proteins were identified using LC–MS on Maxis Impact (Bruker, Billerica, MA, USA).

### 4.7. Transmission Electron Microscopy 

The samples of either full-length *Al*IbpA_His6_ or *Al*IbpAN12_His6_ proteins (cross-linked alone or in presence of substrate proteins) were negatively stained on grids as described in [48]. Briefly, nickel grids (400 mesh, Electron Microscopy Sciences, Hatfield, PA, USA) were treated with 2% collodion solution for film formation. Right before the experiment, grids were discharged using UV during 1 min, followed by immediate addition of 5 μL protein solution for 15 s and its further removal with filter paper. This step was followed by the addition of 4 µL Uranyl Acetate Alternative (Ted Pella, Redding, CA, USA) for 15 s, after which the contrast agent was also removed with filter paper. Samples were visualized on a Libra 120 electron microscope (Zeiss, Oberkochen, Germany) at 10,000–20,000 magnification. The quantification of aggregate sizes was performed using in-house developed software [57].

### 4.8. Bio-Layer Interferometry 

The bio-layer interferometry experiments were performed on the BLItz platform (FortéBio, Fremont, CA, USA) using high-precision streptavidin (SAX) biosensors (FortéBio, Fremont, CA, USA). The protocol provided by BLItz Pro software in the Advanced Kinetics module was modified, as indicated in Supplementary Table S3. 

*Biosensors preparation*. Proteins for immobilization were biotinylated using EZ-Link NHS-PEG4-biotin (Thermo Fisher Scientific, Waltham, MA, USA), as recommended by manufacturer. Briefly, protein solution (10 μM in PBS) was mixed with biotin solution (10 μM in PBS) and incubated for 30 min at room temperature. Free biotin was removed using zebra spin desalting spin columns (3 kDa MWCO) (Thermo Fisher Scientific, Waltham, MA, USA). The streptavidin K biosensors were hydrated in PBS buffer pH 7.4 for 10 min and then loaded into biotinylated protein solution (10 μM) for 120 s. Finally, 2.0 nm protein layer was loaded, and the biosensor was washed with PBS pH 7.4 for 30 s.

*Measurements*. The binding assay was started using equilibration of a prepared biosensor in PBS pH 7.4 for 30 s (baseline). Next, the sensor was loaded into analyte protein solution (10 μM) for 300 s for association and then placed back into PBS for 300 s for dissociation. The biosensors were stored in PBS between measurements. Between samples, the drop holder was washed with 0.1 M HCl, twice-rinsed with pure water and dried with precision wipes (Kimberly Clark, Irving, TX, USA). A 10 μM BSA solution served as negative binding control and was subtracted from the experimental binding data (control curve shown in Supplementary Figure S12). K_D_ values were calculated based on binding-dissociation curves using BLItz Pro data analysis software (version: 1.3.0.5). For each interaction pair, the procedure was repeated at least 6 times in increasing concentrations until the K_D_ value became stable and values were averaged. 

### 4.9. In Vitro Chaperone-like Activity Assay 

The chaperone-like activity was investigated by measuring the capacity of *Al*IbpA to suppress the heat shock-induced aggregation of human insulin (Sigma-Aldrich, St. Louis, MO, USA). The protein aggregation was assessed by measuring the fluorescence of SYPRO Orange, as described in [48]. Briefly, the human insulin (0.1 mg) was mixed with either full-length *Al*IbpA_His6_ or *Al*IbpAN12_His6_ proteins (0.1 mg) in total volume of 30 μL and heated from 10 °C to 95 °C with a temperature increment of 1 °C per 1 min in PBS containing 10 μM SYPRO Orange (Sigma-Aldrich, St. Louis, MO, USA). The fluorescence was measured with excitation at 492 nm and emission at 516 nm on Bio-Rad CFX96 thermocycler [55]. The data were presented as the temperature leading to maximal increase in fluorescent signal (Tm 100).

### 4.10. Cell Viability Test 

The overnight cultures of recombinant *E. coli* strains were diluted in fresh LB medium (1:100) and grown at 37 °C on a shaker at 180 rpm until OD_600_ = 0.6. After 1 h incubation at 30 °C with IPTG (1 mM) to allow proteins expression, the cells were subjected to heat stress (56 °C) for 1 h. Subsequently, 100 µL of yellow tetrazolium salt (3-(4,5-dimethylthiazol-2-yl)-2,5-diphenyltetrazolium bromide, MTT) (Sigma-Aldrich, St. Louis, MO, USA) was added to 100 µL of cell suspension and incubated for 5–10 min at 30 °C. Afterwards, the plates were centrifuged for 5 min at 3500 rpm, supernatant was discarded and 200 µL of DMSO (Merck, Darmstadt, Germany) was added to the sediment to solubilize formazan crystals. The absorbance was measured on a Tecan infinite 200 Pro microplate reader (Tecan, Männedorf, Switzerland) at 550 nm. The viability of cells was calculated in percentage of residual metabolic activity considering the activity before heat treatment as 100%.

### 4.11. Statistical Analyses 

All experiments were performed in biological triplicates with three repeats in each run. The data were analyzed and graphically visualized using GraphPad Prism version 6.00 for Windows (GraphPad Software, Boston, MA, USA, https://www.graphpad.com, accessed on 1 March 2015). The comparison with the control was performed using the non-parametric Kruskal–Wallis test. Significant differences were considered at *p* < 0.05. The elution profiles were analyzed using Waters Breeze 2 software. 

## Figures and Tables

**Table 1:** Amount of *Al*ClpB and *Al*DnaK co-eluting with *Al*IbpA from *A. laidlawii* cell crude extract.

	Score			emPAI		
	4 °C	30 °C	42 °C	4 °C	30 °C	42 °C
*Al*DnaK	465	1508	1412	0.47	0.73	0.83
*Al*ClpB	222	532	11,703	0.09	0.31	2.72

## Data Availability

The data analyzed during the current study are available from the corresponding author upon request.

## Supplementary Materials

The supporting information can be downloaded at: https://www.mdpi.com/article/10.3390/ijms242015445/s1. References [58,59] are cited in the supplementary materials.
