# Supplementary material for: The Functionality of IbpA from Acholeplasma laidlawii Is Governed by Dynamic Rearrangement of Its Globular–Fibrillar Quaternary Structure
Source: Int J Mol Sci. 2023 Oct 22;24(20):15445. doi: 10.3390/ijms242015445 (PMC10607609; doi:10.3390/ijms242015445)
Supplement: Supplementary file 1 [file ijms-24-15445-s001.zip › ijms-2591499-supplementary.pdf]

Supplementary Material

Protein purification

E. coli BL21(DE3) cells were transformed with plasmids shown in Supplementary Table S3. The cells were grown at 37 °C in 500 ml of LB medium supplemented with 100 µg ml^{-1} ampicillin. After growth of the culture to an optical density (OD_{600}) of 0.8, protein expression was induced by the addition of IPTG (until final concentration 1 mM). Four hours after induction, cells were harvested by centrifugation and stored at −20 °C.

Proteins with N-terminal His6-tag were purified using affinity chromatography on Ni-NTA sepharose gravity-flow columns (Qiagen, Hilden, Germany) following the protocol described previously [48]. *E. coli* cell-free extracts were prepared from the frozen cells (see above) by resuspending the cells in disruption buffer DB [50 mM Tris–HCl pH 7.4, 50 mM KCl, 500 mM NaCl, 5 mM $MgCl_2$, 1 mM EDTA, 2 mM benzamidine, 1 mM phenylmethylsulfonyl fluoride (PMSF)] and broken by sonication. Unbroken cells and debris were removed after 10 min centrifugation at 8000 rpm. The cell-free extracts were centrifuged again for 20 min at 15000 rpm. The supernatant was passed through a 1 ml His-select cartridge column that was pre-equilibrated with washing buffer (50 mM Tris–HCl pH 8.0, 500 mM NaCl, 10 mM imidazole) and unbound proteins were washed out with 15 ml washing buffer. Finally, the affinity-bound proteins were eluted with 8 ml elution buffer (50 mM Tris–HCl pH 8.0, 500 mM NaCl, 500 mM imidazole).

Proteins with C-terminal StrepII-tag were purified using affinity chromatography on Strep sepharose gravity-flow columns (Qiagen, Hilden, Germany) following the protocol described previously [57]. *E. coli* cell free extracts were prepared from the frozen cells (see above) by resuspending the cells in Strep-wash buffer [100 mM Tris–HCl pH 8, 150 mM NaCl, 1 mM EDTA, 2 mM benzamidine] and broken by sonication. Unbroken cells and debris were removed after 10 min centrifugation at 8000 rpm. The cell-free extracts were centrifuged again for 20 min at 15000 rpm. The supernatant was passed through a 1 ml Strep-select cartridge column that was pre-equilibrated with regeneration buffer (HABA) and unbound proteins were washed out with 15 ml washing buffer. Finally, the affinity-bound proteins were eluted with 8 ml elution buffer (100 mM Tris–HCl pH 8.0, 150 mM NaCl, 1 mM EDTA, 2.5 mM Desthiobiotin (100x)).

All proteins were purified to apparent electrophoretic homogeneity and dialyzed against PBS pH 7.4 containing 100 mM NaCl and used immediately for experiments or stored in ice no more than 4 days. Protein concentration was measured with the Roti-Quant protein assay reagent (Carl Roth, Karlsruhe, Germany) and bovine serum albumin (Carl Roth, Karlsruhe, Germany) as a standard.

Supplementary Table S1. Primers used in this study

Primer name	Primer sequence 5'-3'	Description
**IbpA L for**	5' TAA TCC GCA GGG TAC ACA AAT CG 3'	For *EcibpA/B* gene deletion
**IbpA L rev**	5' CAG TCT AGC TAT CGC CAT GTA AGC CCT CAG GTA GCC AGA ACA CCC ATC AG 3'	For *EcibpA/B* gene deletion
**IbpB L rev**	5' CAG TCT AGC TAT CGC CAT GTA AGC CCC AGG CGG CCT TAG GGA ATT AGT TG 3'	For *EcibpA/B* gene deletion
**IbpA R for**	5' CTG CCA TCA CGA GAT TTC GAT TCC CAA CTA ATT CCC TAA GGC CGC CTG 3'	For *EcibpA/B* gene deletion
**IbpB R for**	5' CTG CCA TCA CGA GAT TTC GAT TCC ACG TTC TTT CCC GTG ATA ATG TGC 3'	For *EcibpA/B* gene deletion
**IbpB R rev**	5' ATG GCT AAA TCA TGC AGG TTA GGG 3'	For *EcibpA/B* gene deletion
**Km for**	5' GGG CTT ACA TGG CGA TAG CTA GAC TG 3'	For *EcibpA/B* gene deletion
**Km rev**	5' GGA ATC GAA ATC TCG TGA TGG CAG 3'	For *EcibpA/B* gene deletion
**IbpA seq for**	5' CTG ATG GGT GTT CTG GCT ACC TGA C 3'	For *EcibpA/B* gene deletion
**IbpA seq rev**	5' GCA CAT TAT CAC GGG AAA GAA CGT G 3'	For *EcibpA/B* gene deletion
**ClpB L for**	5' CGT AGG AGT CTG GAC CGT GTC TCA G 3'	For *EcclpB* gene deletion
**ClpB L rev**	5' CGT TTG TTG AAC TAA TGG GTG CCG CTT GTC AGG CCG GAA TAA C 3'	For *EcclpB* gene deletion
**ClpB R for**	5' CGT CCG GCG TAG AGG ATC TGG AGC GCT ACT GGA GAT TAA ATG AGG 3'	For *EcclpB* gene deletion
**ClpB R rev**	5' GAC TGT GCG CAG GCG TGC TG 3'	For *EcclpB* gene deletion
**Cm for**	5' GCA CCC ATT AGT TCA ACA AAC G 3'	For *EcclpB* gene deletion

**Cm rev**	5’ CGT CCG GCG TAG AGG ATC TGG AGC 3’	For *EcclpB* gene deletion
**dnaK L for**	5’ GTT CGT TGC GTA TCA GTA CGC TGG AGA GGC 3’	For *EcdnaK* gene deletion
**dnaK L rev**	5’ CAA TTT ACT TAC CCT AGA TTG GAC GCA GAC TCA CTC ACT GCG GTT GAC TAC 3’	For *EcdnaK* gene deletion
**dnaK R for**	5’ GCG CAT TCC CGA TGA AGA GGC CGG CGC CTG GCC ATG AAA TAC CAC CCG GAC CG 3’	For *EcdnaK* gene deletion
**dnaK R rev**	5’ GCC TAG AAA CTA GTC GCA TGG C 3’	For *EcdnaK* gene deletion
**Spc for**	5’ GTC CAA TCT AGG GTA AGT AAA TTG 3’	For *EcdnaK* gene deletion
**Spc rev**	5’ GCG CAT TCC CGA TGA AGA GGC CGG 3’	For *EcdnaK* gene deletion

Supplementary Table S2. Plasmids used in the study

Plasmid	Description	Source
pET15b	Expression vector, IPTG- inducible	Novagen, Madison, Wisconsin, USA
pet-*Al*IbpA$_{His6}$	Overexpression of His-tagged IbpA in *E. coli*	[47]
pet-*Al*IbpAΔN12$_{His6}$	Overexpression of His-tagged IbpA lacking 12 amino acid residues from the N-termini	[48]
pET-28a-*Al*DnaK$_{\mathbf{ST}}$	Overexpression of StrepII-tagged DnaK in *E. coli*	Synbio Technologies, Monmouth Junction, USA
pET-28a-*Al*ClpB$_{ST}$	Overexpression of StrepII-tagged ClpB in *E. coli*	Synbio Technologies, Monmouth Junction, USA
pKT25	for amplification of the kanamycin resistance gene	Euromedex, Souffelweyersheim, France)
pDG1661	for amplification of the spectinomycin resistance gene	[58]
pDG1661	for amplification of the chloramphenicol resistance gene	[58]
pSIM5-tet	λ Red recombination plasmid, temperature-inducible; TcR	[59]

Supplementary Table S3. Biolayer interferometry measurement protocol for protein-protein interactions.

Step type	*Sample type*	*Duration (s)*	*Position*
Initial baseline	PBS buffer	30	Tube
Loading	Protein	120	Drop
Baseline	PBS buffer	30	Tube
Baseline	PBS buffer	30	Tube
Association	Protein or protein complex	300	Drop
Dissociation	PBS buffer	300	Tube

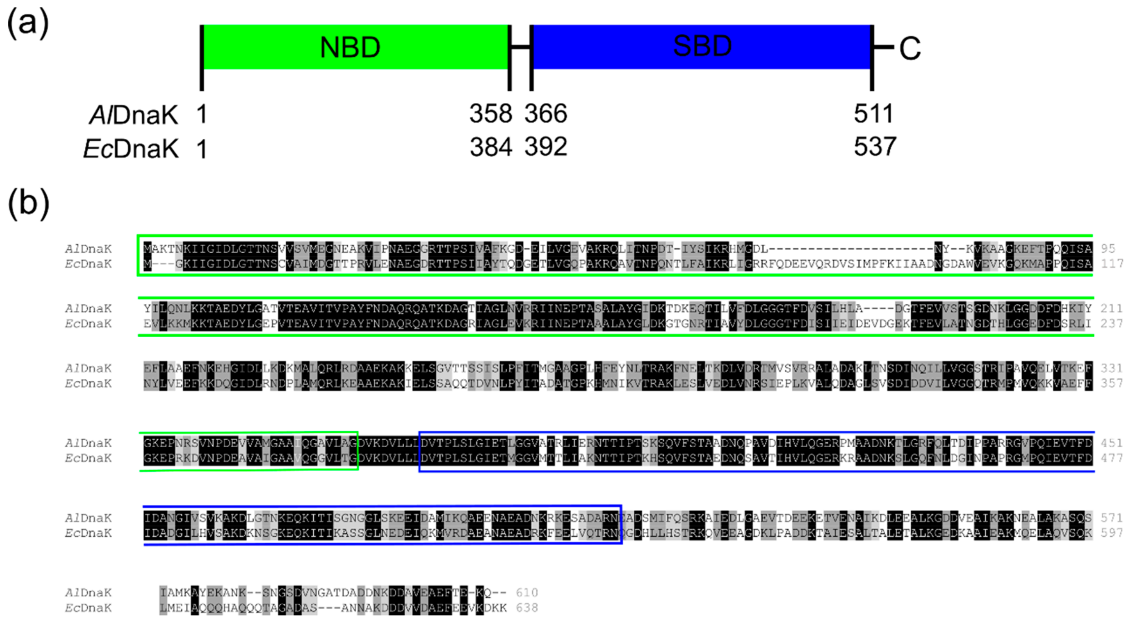

Supplementary Figure S1. Domain organization of the heat shock protein 70 (HSP70, DnaK) system in *E. coli* (*Ec*DnaK) and *A. laidlawii* (*Al*DnaK) **(a)** Scheme of HSP70 domain organization. NBD: nucleotide-binding domain; SBD: substrate-binding domain; C-terminus. Numeration is according to the HSP70 from *Acholeplasma laidlawii* PG-8A ABX81168.1 for *Al*DnaK and *Escherichia coli* NP_414555.1 for *Ec*DnaK. The numbers below the scheme show the first and the last amino acid of each domain. **(b)** Alignment of amino acid sequences of HSP70 from *A. laidlawii* (*Al*DnaK) to *E.coli* (*Ec*DnaK). Amino acids in black represent identical residues, homologous substitutions are shown in grey. Functional motifs are in frames.

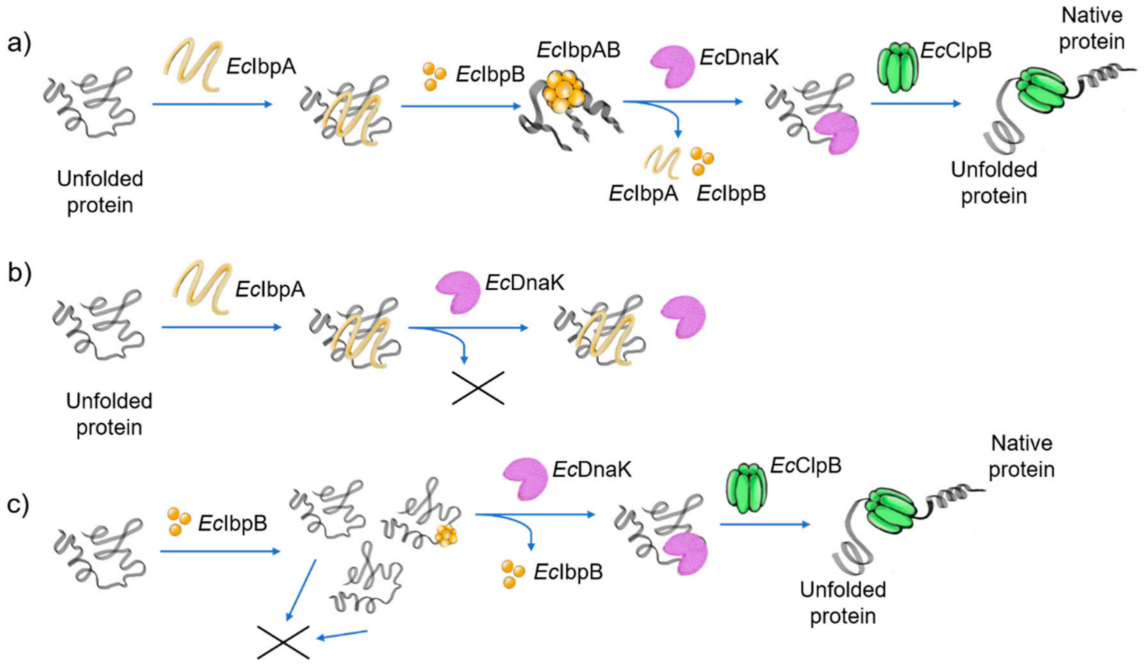

Supplementary Figure S2. Described HSP20-70-100 (IbpA/B-DnaK-ClpB) mediated protein disaggregation mechanism in *E. coli:* **(a)** in presence of both components of multi-chaperone system and **(b)** absence of *Ec*IbpB or **(c)** *Ec*IbpA [43, 44, 52].

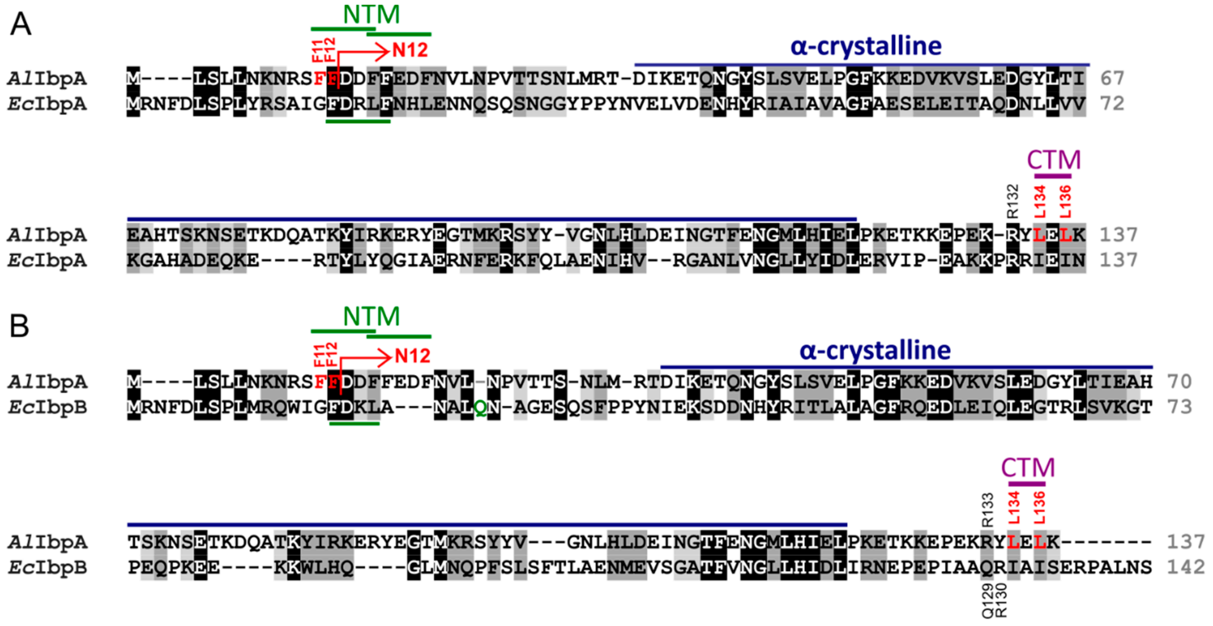

C

	N-terminus		C-terminus	
	Identity, %	Similarity, %	Identity, %	Similarity, %
***Ec*IbpA**	18	37	27	53
***Ec*IbpB**	25	38	0	26

Supplementary Figure S3. The alignment of amino acid sequences of IbpA from *A. laidlawii* (*Al*IbpA) to IbpA/B from *E.coli*: *Ec*IbpA **(A)** or *Ec*IbpB **(B)**. Amino acids in black represent identical residues, homologous substitutions are shown in grey. Functional motifs are underlined. NTM: N-terminal putative motif (W/F)(D/F) PF; α-crystalline; CTM: C-terminal motif V/IXI/V; N-terminal truncation in *Al*IbpA is shown with arrow. **(C)** The identity and similarity of N-termini and C-termini of *Al*IbpA with those of HSP20 proteins IbpA and IbpB from *E. coli*.

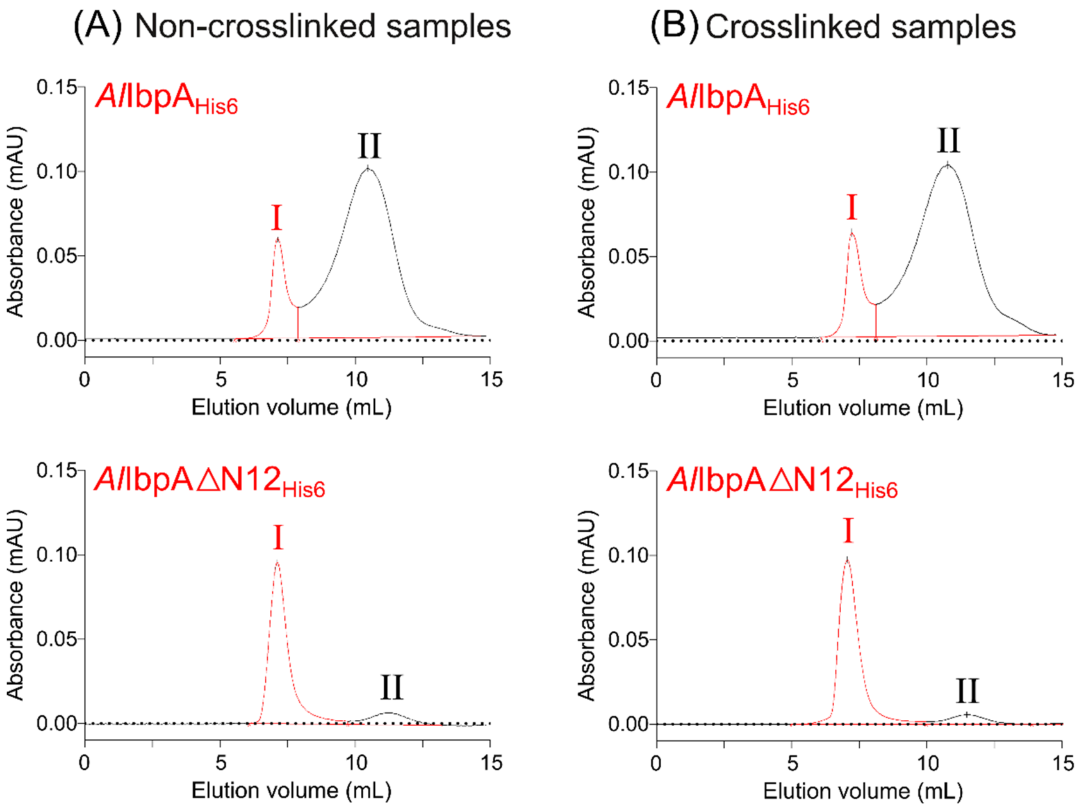

Supplementary Figure S4. The oligomerization state of native (A) or chemically cross-linked (B) recombinant full-length *Al*IbpA$_{His6}$ and N-terminally truncated *Al*IbpAΔN12$_{His6}$ analyzed with size-exclusion chromatography. I and II indicate specific elution peaks.

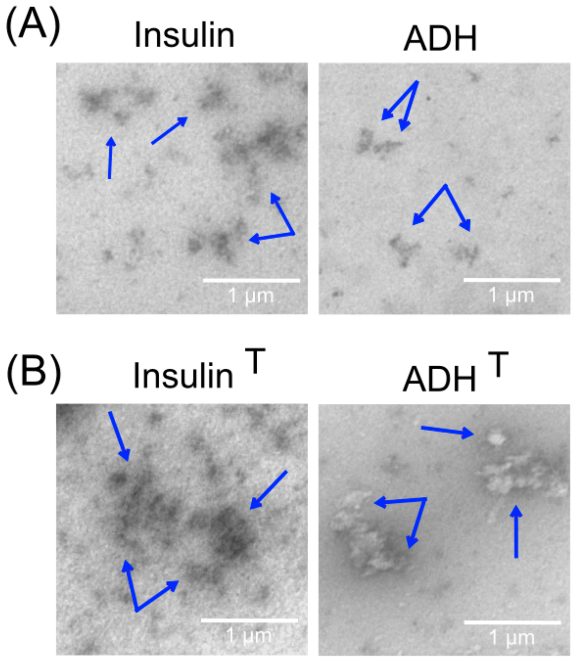

Supplementary Figure S5. The conformation of native **(A)** and thermally inactivated **(B)** insulin and alcohol dehydrogenase (ADH). Proteins were preincubated for 30 min at 25 °C **(A)** or 56 °C **(B)**, treated with glutaraldehyde and analyzed with transmission electron microscopy. Blue arrows indicate protein aggregates.

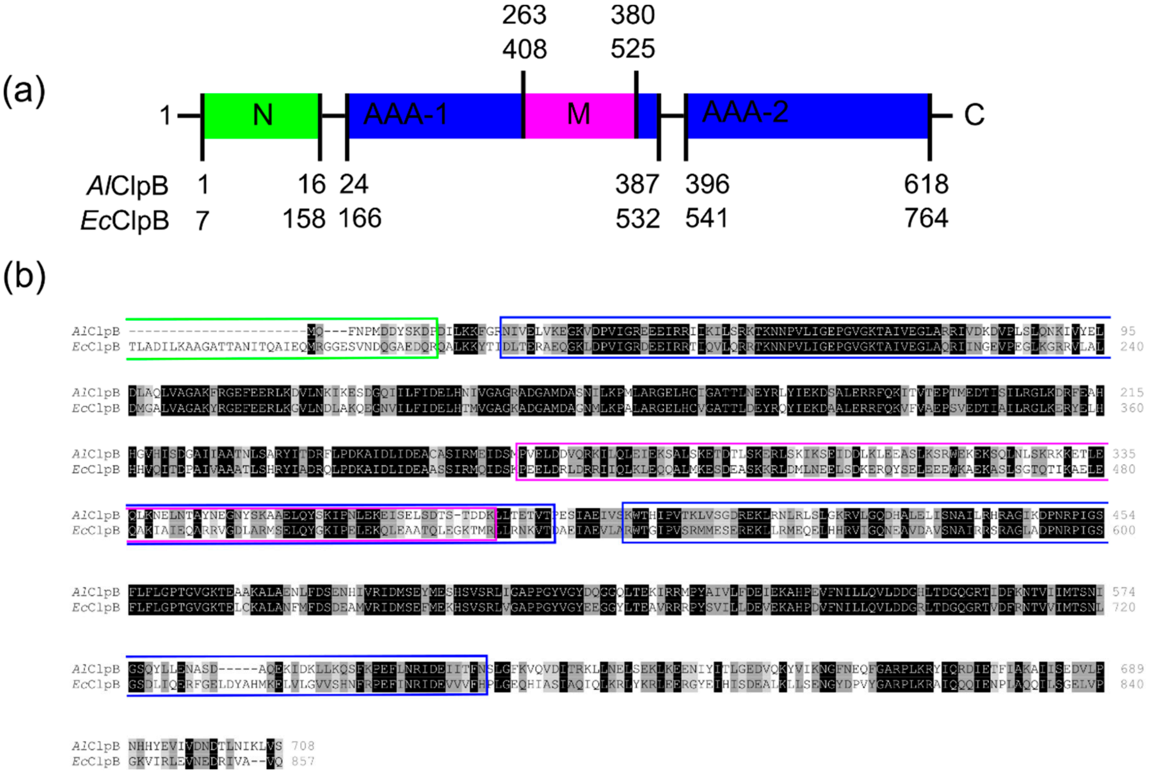

Supplementary Figure S6. Domain organization of HSP100 (ClpB) from *E. coli* (*Ec*ClpB) and *A. laidlawii* (*Al*ClpB). **(a)** Scheme of HSP100 domain organization. NBD: substrate-binding and recognition domain; two nucleotide-binding domains (AAA-1 and AAA-2) and middle domain (M); C-terminus. Numeration is according to ClpB from *Acholeplasma laidlawii* PG-8A ABX81733.1 for *Al*ClpB and *Escherichia coli* AAC75641.1 for *Ec*ClpB. The numbers below the scheme show only the first and the last amino acid of each domain. **(b)** Alignment of amino acid sequences of *Al*ClpB and *Ec*ClpB. Amino acids in black represent identical residues, homologous substitutions are shown in grey. Functional motifs are in frames.

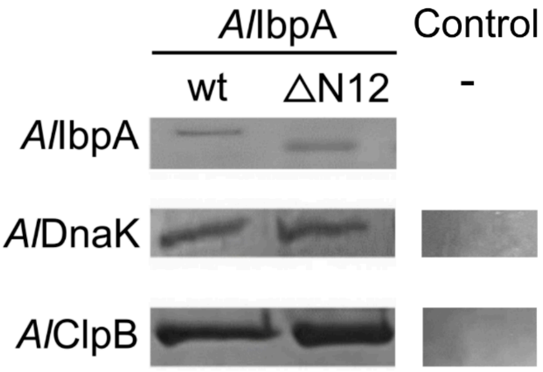

Supplementary Figure S7. *In vitro* interaction of purified full-length His6-tagged *Al*IbpA (wt) or N-terminally truncated *Al*IbpAΔN12$_{His6}$ (ΔN12) with StrepII-tagged *Al*DnaK or *Al*ClpB was followed by pull-down of interacting proteins using Ni-NTA beads. Proteins were mixed in PBS in a 1:1 (w/w) ratio and after 30 min of incubation at 25 °C were purified on Ni-NTA agarose. The elution fractions were separated using 15% PAGE under denaturing conditions. Coelution without *Al*IbpA$_{His6}$ served as negative control.

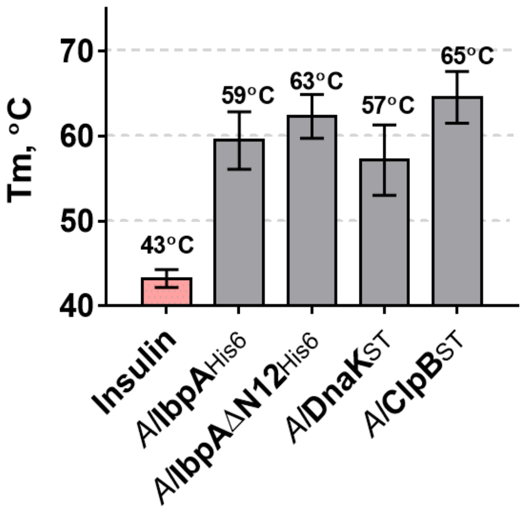

Supplementary Figure S8. Tm values of human insulin and HSPs *in vitro*. Various proteins (0.1 mg) in total volume of 30 µl were heated from 10 °C to 95 °C with a temperature increment of 1 °C per 1 min in the presence of 10 µM SYPRO Orange. The fluorescence was detected by using FAM-filter set detection on Bio-Rad CFX96 thermocycler. The data analysis was performed by determining the temperature leading to fluorescence increase by 100% of maximal signal (T_m).

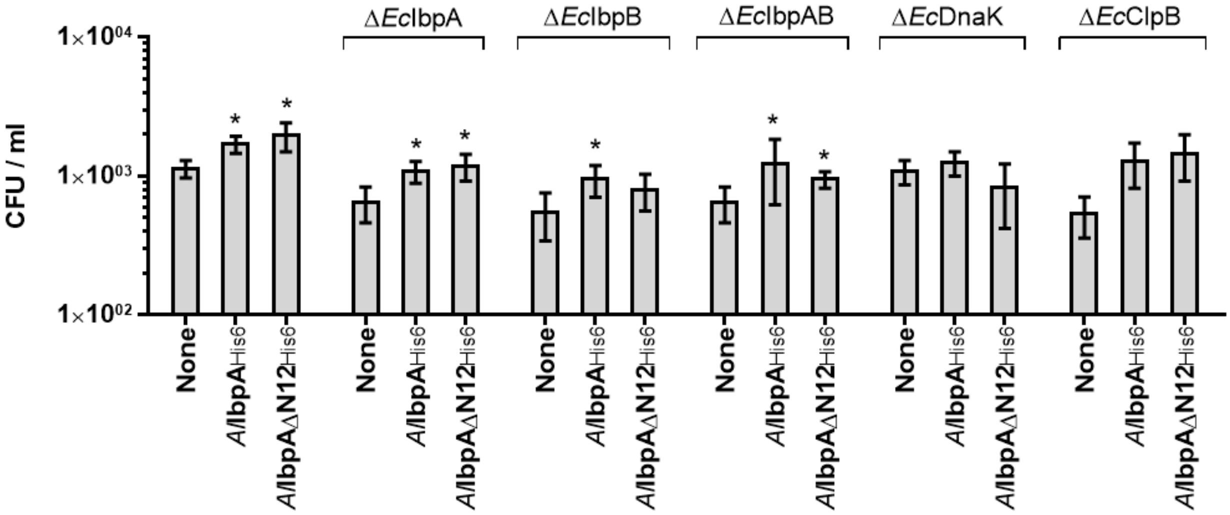

Supplementary Figure S9. The viability of *E. coli* BL21 and its HSP-deficient derivatives ectopically overexpressing either full length or N-terminally truncated *Al*IbpA. Cells carrying pET15b vector without insert served as a control (marked as "None"). Exponentially grown cells were induced with 1 mM IPTG. After 2 h of growth at 30 °C, cultures were heat-treated (1 h at 56 °C) and residual viability was assessed in colony forming units (CFUs) count assay. Data are present as mean ± standard deviations of biological triplicates with 3 technical repeats of each. Asterisks show statistically significant differences between mean values of cells with and without sHSP overexpression ($p < 0.05$).

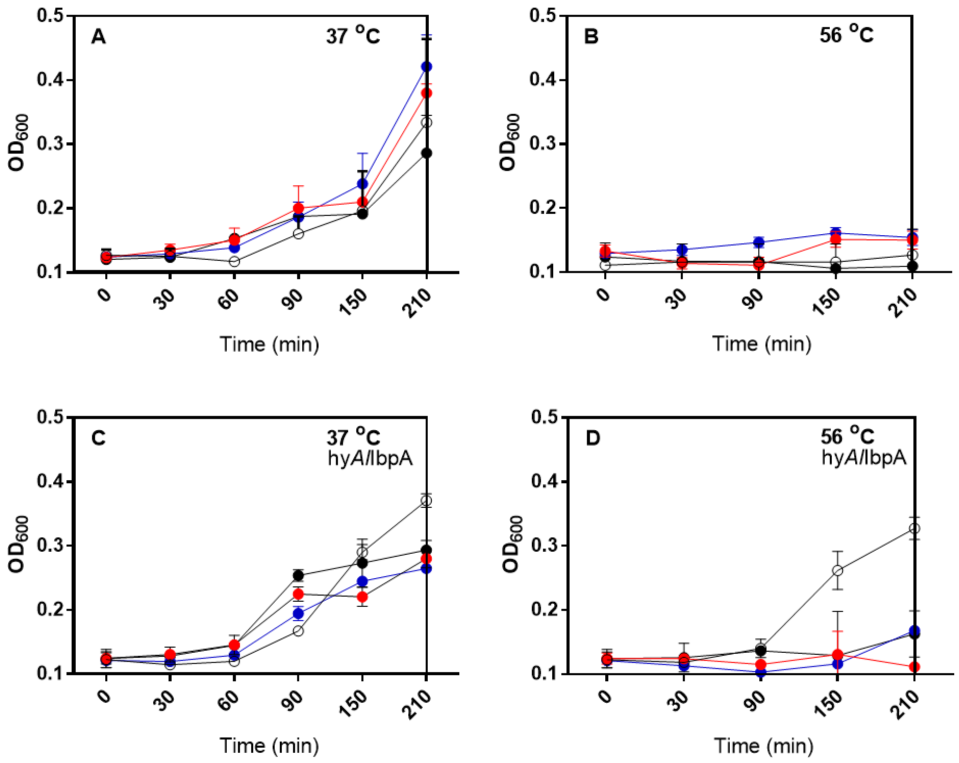

Supplementary Figure S10. Growth of *E. coli* BL21 wt (open circle) and *E. coli* BL21 ΔHSPs: ΔIbpA (red circle); ΔIbpB (blue circle); ΔIbpAB (black circle). **(A)** Growth at 37 °C; **(B)** Growth at 56 °C; **(C-D)** Growth of *E. coli* with *Al*IbpA overproduction. The strains were cultured as biological triplicates in LB medium. Standard deviations of the mean were plotted as error bars. The measurement of optical density at 600 nm (OD_{600}) was used to determine the stage of growth of the *E. coli* BL21 culture.

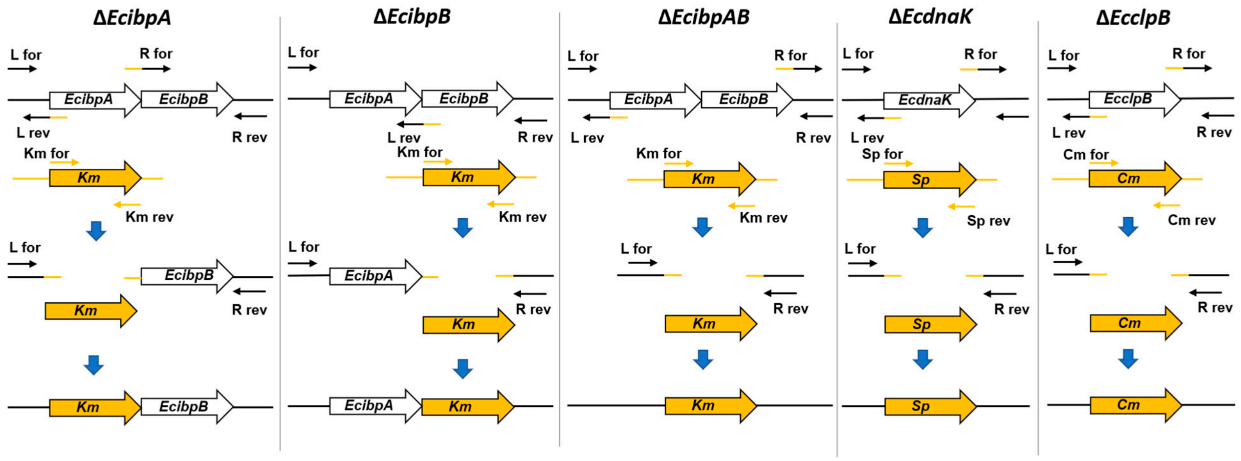

Supplementary Figure S11. Genetic constructs for HSP knockout in *E. coli* cells. Antibiotic resistance cassettes used for substitution with the genes of interested are colored in yellow (Km: Kanamycin; Sp: Spectinomycin; Cm: Chloramphenicol).

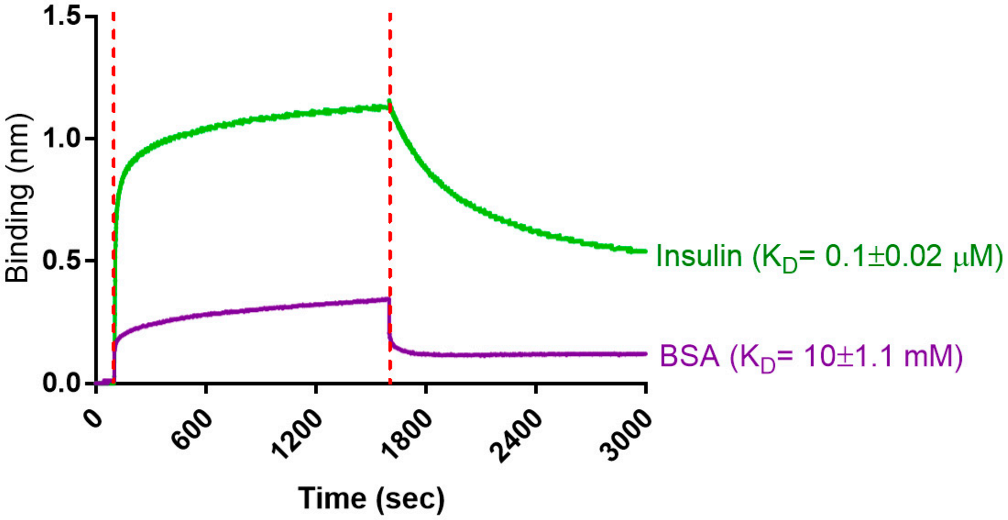

Supplementary Figure S12. The interferometric analysis of recombinant *Al*IbpA$_{His6}$ interaction with insulin and BSA (bovine serum albumin) *in vitro* on the BLItz instrument (FortéBio, Fremont, CA, USA). *Al*IbpA$_{His6}$ was loaded onto streptavidin biosensor until 2.0 nm protein was bound to the surface, after 30 sec washing in PBS the sensor was immersed into either insulin or BSA solutions (10 μM). A dissociation constant (K_D) calculation was performed with BLItz Pro data analysis software (version: 1.3.0.5). The standard deviations from technical triplicates are indicated as range behind calculated K_D mean values.
